# Inter-annual growth of Arctic charr (*Salvelinus alpinus*, L.) in relation to climate variation

**DOI:** 10.1186/1472-6785-6-10

**Published:** 2006-08-27

**Authors:** David M Kristensen, Thomas R Jørgensen, Rasmus K Larsen, Mads C Forchhammer, Kirsten S Christoffersen

**Affiliations:** 1 Dept. of Medical Biochemistry and Genetics A, Panum Institute, University of Copenhagen, Blegdamsvej 3, DK-2200 Copenhagen, Denmark; 2 Dept. of Veterinary Pathobiology, Royal Veterinary and Agricultural University, Stigbøjlen 4, DK-1870 Frederiksberg C, Denmark; 3 Freshwater Biological Laboratory, Institute of Biology, University of Copenhagen, Helsingørsgade 51, DK-3400 Hillerød, Denmark; 4 Dept. of Arctic Environment, National Environmental Research Institute, Frederiksborgsvej 399, P.O. Box 358, DK-4000 Roskilde, Denmark

## Abstract

**Background:**

Major changes in climate have been observed in the Arctic and climate models predict further amplification of the enhanced greenhouse effect at high-latitudes leading to increased warming. We propose that warming in the Arctic may affect the annual growth conditions of the cold adapted Arctic charr and that such effects can already be detected retrospectrally using otolith data.

**Results:**

Inter-annual growth of the circumpolar Arctic charr (*Salvelinus alpinus*, L.) was analysed in relation to climatic changes observed in the Arctic during the last two decades. Arctic charr were sampled from six locations at Qeqertarsuaq in West Greenland, where climate data have been recorded since 1990. Two fish populations met the criteria of homogeny and, consequently, only these were used in further analyses. The results demonstrate a complex coupling between annual growth rates and fluctuations in annual mean temperatures and precipitation. Significant changes in temporal patterns of growth were observed between cohorts of 1990 and 2004.

**Conclusion:**

Differences in pattern of growth appear to be a consequence of climatic changes over the last two decades and we thereby conclude that climatic affects short term and inter-annual growth as well as influencing long term shifts in age-specific growth patterns in population of Arctic charr.

## Background

The number of Arctic charr (*Salvelinus alpinus*, L.) phenotypes varies considerably within and across localities on Disko Island off the west coast of Greenland [1,2]. Two landlocked populations have been described here as well as dimorphic populations with an anadromous morph and a resident morph in rivers [1,2], but whether the two morphs are reproductively isolated remain unclear. The resident and landlocked populations are isolated in the same locality throughout their entire life cycle, thereby presenting an excellent opportunity to investigate differential effects of abiotic factors on somatic growth.

Fish are ectotherms and hence rely exclusively on external sources of heat. Metabolic produced heat is rapidly lost through the gills and the epidermis and consequently body temperature fluctuates closely with changes in the ambient water temperature [3]. Indeed, since the metabolic processes within the animals are strongly temperature dependent [4], ambient water temperature exerts a major influence on all physiological and behavioural processes in the fish. Moreover, temperature has a non-linear effect on physiological responses, with an identifiable optimum-rate, and it has been argued that the thermal niche of a fish should considered in much the same way as for example a food resource [5]. Also, it has been shown that that ovulation in charr markedly decreases above 8°C and is inhibited at 10°C [6]. Finally, maintaining fish above 5°C for several weeks results in reduced quality of the eggs [7,8]. Hence, relatively small absolute changes in ambient temperature may induce profound changes in the life history traits of the Arctic charr.

Major changes in climate have already been observed in the Arctic and climate models predict further amplification of the enhanced greenhouse effect at high-latitudes leading to increased warming [9,10]. In the present study, we propose that warming in the Arctic may affect the annual growth conditions of the cold adapted Arctic charr and that such effects can already be detected retrospectrally using otolith data. To evaluate this hypothesis in 2004 we sampled fish and otoliths from populations at several localities at Disko Island, where the local climate shows an increasing trend in temperatures in the period 1990 to 2004 [10]. Finally, we compare the age specific growth in 2004 with age specific growth data sampled in 1990 [2].

## Results

### Description of the two homogenous and landlocked populations

The investigations of population structure using Principal Component Analysis (PCA) showed that only one morph inhabited the landlocked localities Røde Elv (69° 16'N, 53° 29'W) and Kangarssuk (69° 16'N, 53° 50'W) [see Additional file 1]. Therefore, only individuals sampled from these two populations were used in the analysis. All individuals from both localities had parr marks characteristic of fish that have not smoltificated. The individuals from Røde Elv were small, ranging in size from 82 to 169 mm, and had large eyes and ventrally placed mouths. Despite their small size, a number of fish in Røde Elv were ready to spawn, a feature common to both populations. Specific for all fish sampled at Kangarssuk were large external, infectious wounds. Fish in Kangarssuk were large, ranging in size from 220 to 367 mm and a considerable number were ready to spawn. All individuals caught at Kangarssuk were more than 5 years old, while ages of individuals in other populations were uniformly distributed. All had eaten chironomids and additionally a large number of individuals had preyed on benthic ostracods and *Lepidurus arcticus*.

### Annual fish growth rate and effects of climate variation

Detailed results from multiple linear regressions are depicted in table 1. Regressions were carried out with growth rate residuals (*GR*_*t*_) as response variable and one year delayed autoregressing (*GR*_*t*-1_), mean annual temperature (T_a_), mean summer temperature (T_s_), mean winter temperature (T_w_) and mean annual precipitation (P_a_) as predictor variables. None of the four weather parameters showed significant interdependence. Analyses of cohorts caught in 2004 were based on weather data from Qeqertarsuaq, while analyses from 1990 cohorts were carried out using data from Aasiaat.

**Figure 1:**
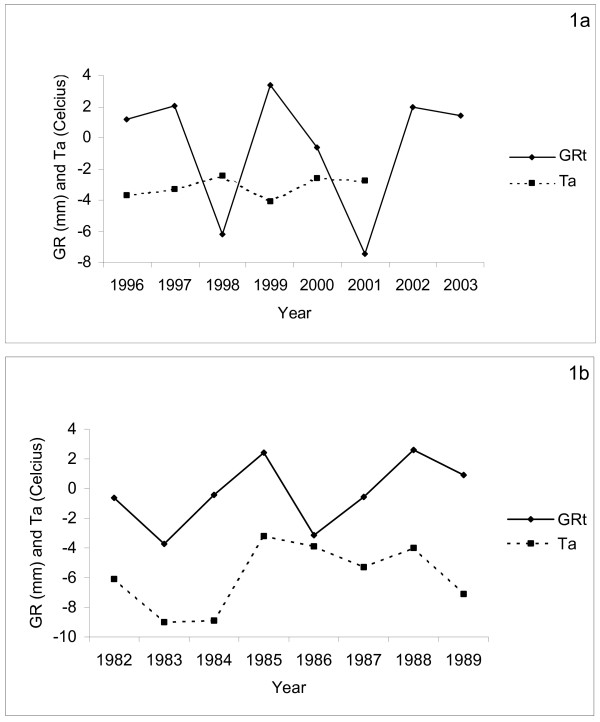
**Growth rates obtained from otoliths of Arctic charr caught in Røde Elv 2004 and 1990 plotted against fluctuations in mean annual temperature**. (a) Data from 2004 showing *GR*_*t *_(*GR*_*t *_= Growth rate residuals) in Arctic charr from Røde Elv plotted against fluctuations in T_a _(T_a _= Mean annual temperature). (b) Data from 1990 showing *GR*_*t *_in Arctic charr from Røde Elv plotted against fluctuations in T_a _data from Aasiaat.

Analyses of the Røde Elv individuals caught in 2004 (n = 14) showed a negative effect of T_a _on the annual *GR*_*t *_together with a non-significant *GR*_*t*-1 _(R^2 ^_total _= 0.92; T_a_: p = 0.05; *GR*_*t*-1_: p = 0.11; Fig 1a). The otolith data from this sample allow regression with more than two predictor variables in only two cases but neither yielded any significant results (table 1). Otolith data from individuals caught in Røde Elv in 1990 (n = 14) by contrast, allowed simultaneous modelling of several predictor variables and *GR*_*t*_. These data showed a significant positive relationship with T_a _and a significant negative relationship with P_a_(R^2^_total _= 0.83; T_a_: p = 0.03; P_a_: = 0.05; *GR*_*t*-1_: p = 0.78; Fig 1b).

**Figure 2:**
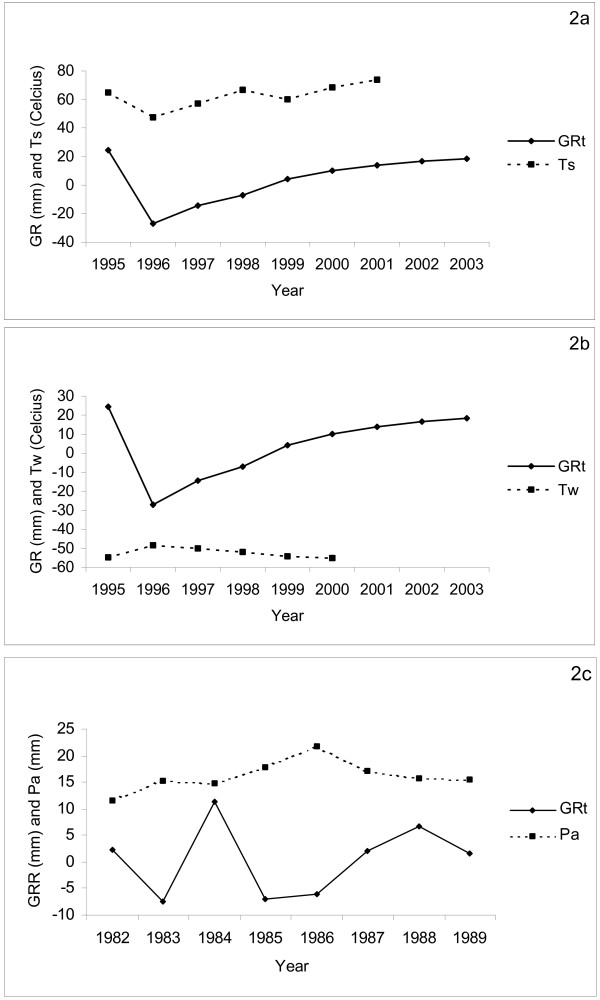
**Growth rates obtained from otoliths of Arctic charr caught in Kangarssuk 2004 and 1990 plotted against fluctuations in mean annual temperature and precipitation**. (a) Data from 2004 showing *GR*_*t *_(*GR*_*t *_= Growth rate residuals) in Arctic charr from Kangarssuk plotted against fluctuations in T_s _(T_s _= Mean summer temperature) (b) and T_w _multiplied by 5 (T_w _= Mean winter temperature). (c) Data obtained from otoliths collected in 1990 showing *GR*_*t *_in Arctic charr from Kangarssuk plotted against fluctuations in P_a _divided by 20 from Aasiaat (P_a _= Mean summer temperature).

Data on annual *GR*_*t *_from the Kangarssuk individuals caught in 2004 (n = 14) allowed simultaneous multiple linear regression with several predictor variables and the analysis yielded several significant relationships between *GR*_*t *_and T_s _and T_w_, respectively (se Fig 2a, 2b and table 1). For example, multiple linear regression with *GR*_*t *_and the predictor values T_s_, T_a _and *GR*_*t*-1 _showed a positive significant connection (R^2^_total _= 0.94; T_s_: p = 0.04; T_a_: p = 0.18; *GR*_*t*-1_: p = 0.66), while a similar regressing with *GR*_*t *_and the predictor variables T_w_, T_a _and *GR*_*t*-1 _showed a negative significant relationship (R^2^_total _= 0.98; T_w_: p = 0.04; T_a_: p = 0.81; *GR*_*t*-1_: p = 0.36). Thus, *GR*_*t *_are significantly related negatively to T_w _and positively to T_s_, without any significant *GR*_*t*-1_.

Data on annual *GR*_*t *_from individuals caught in Kangarssuk in 1990 (n = 10) allowed multiple linear regressions incorporating several predictor variables. A model incorporating T_a_, P_a _and *GR*_*t*-1 _showed a negative effect of P_a _on *GR*_*t *_(R^2^_total _= 0.77; P_a_: = 0.07; T_a_: p = 0.21; *GR*_*t*-1_: p = 0.09), while a model incorporating T_w _instead of T_a _further documented the relationship (R^2^_total_= 0.8; P_a_: = 0.07; T_w_: p = 0.17; *GR*_*t*-1_: p = 0.09; se also Fig 2c).

### Comparison of mean growth rate from the two time series

**Figure 3:**
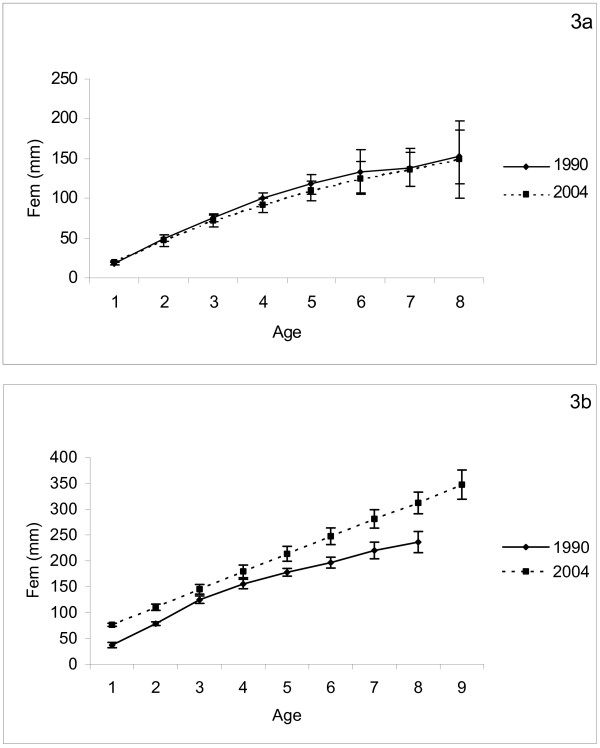
**Comparison of growth rates of Arctic charr from Kangarssuk and Røde Elv sampled in 1990 and 2004**. (a) Changes in values of fork length estimated means showing growth during life history of fish living at Røde Elv in 1982–1989 and 1995–2003 (F_em _= Values of fork length estimated means; error bars: ± 95% C.L; ANOVA: p = 0.008; d.f. = 7) and (b) at Kangarssuk in respectively 1982–1989 and 1995–2003 (F_em _= Values of fork length estimated means; Error bars: ± 95% C.L; ANOVA: p = 0.0004; d.f. = 7).

Otolith derived changes in mean values of fork length estimated means (F_em_), and thereby growth, were assessed from the periods 1982–1989 and 1995–2003, respectively. An ANOVA test was performed to evaluate the differences between the time series. The estimated rates of growth from the Røde Elv samples from 1982–1989 were significantly larger compared with the 1995–2003 sample (p = 0.008; d.f. = 7; Fig 3a). The opposite was the case among fish from Kangarssuk. Here, rates of growth were larger in the period 1991–2003 compared to 1982–1989 (p = 0.0004; d.f. = 7; Fig 3b).

## Discussion

Our findings suggest that the climatic variation observed in the Arctic during the last two decades has already had a significant effect on life history parameters in Arctic charr. This is evident from the complex coupling between annual growth rates and fluctuations in mean annual temperatures and means annual precipitation together with marked changes in the annual mean growth rate history between 1990 and 2004. Impacts of climate on animal populations have been demonstrated before for several species in the Arctic e.g. musk oxen, reindeer, vole and wolf [11]. These species are generally morphologically and ecologically more uniform than Arctic charr which is one of the most polymorphic vertebrate species [12]. Consequently, several difficulties would be anticipated addressing the modelling analyses without consideration for known variation in life history and hence somatic growth rates for different morphs – e.g. anadromous versus residents. The homogenous and landlocked Arctic charr in Røde Elv and Kangarssuk were therefore the only suitable populations to study annual climatic impact on growth of an Arctic population. The mean air temperatures in August at sample sites have increased from 4.9°C in 1991 to 9.1°C in 2001 [10]. Consistent with our prediction this appears to have retarded the rate of growth of fish in one population (Røde Elv) but, contrary to our expectations to have stimulated annual rates of growth in another (Kangarssuk). We suggest that the difference in response might be due to local differences in the dynamic between energy demand in fish and food supply.

An increase in ambient temperature leads to increase in metabolic rate, which in turn leads to an increased energy demand. If increased metabolic demand is not matched by a corresponding increase in food supply, the net outcome is likely to be a reduction in fitness and, hence, growth rate of the fish [13]. Røde Elv already is a poor habitat for charr, as indicated by the small maximum size of fish there (16.9 cm), and higher temperatures may therefore have resulted in an increased metabolic demand which the fish were unable to adequately meet. At Kangarssuk, in contrast, there appear to have been an increase in food supply evidenced by the appearance of benthic ostracods and *Lepidurus arcticus *as new food items in 2004 compared to prior sampling at the locality [2].

After showing climate dependent growth rates in fish caught in both 1990 and 2004, the time series were compared. The data demonstrated a difference between F_em _from the 1990 and 2004 time series at both Røde Elv and Kangarssuk. The charr population in Røde Elv in 2004 had experienced a decline in F_em _compared to 1990. As mentioned, several factors such as elevated temperatures, increased turbidity or difference in prey composition may explain this tendency. The definition of optimum temperature for growth is only valid under the assumption that there is no food limitation [14]. Hence, the temperature at which the growth rate is maximised is progressively shifted to lower temperature values, as the amount of available food is decreased [13]. Our data from Røde Elv is in accordance with the above mentioned scenario showing a significant positive influence of T_a _on the population sampled in 1990, while samples from 2004 show a significant negative influence of T_a _on the annual growths rate. Thus, changes in F_em _between 1990 and 2004 supports our hypothesis that in Røde Elv energy resources cannot sustain higher metabolic rates created by increasing temperatures. Consequently, there has been a drop in fitness mirrored in the reduced growth rate.

From the Kangarssuk data it is evident that F_em _values are considerably larger in the 2004 compared to 1990. Furthermore, all individuals caught in Kangarssuk in 2004 had large skin infections, which were not observed in 1990 [2]. Several studies have shown that parasitic, viral and bacterial infections in fish are positively correlated to ambient temperatures [15-17]. It seems therefore reasonable that the infections could be a consequence of the increase temperature in the lake. In addition, we did not catch any juvenile individuals in Kangarssuk, which is also contrary to studies in 1990 [2] and the other sampled localities. The fact that it was impossible to catch fish below age 5+ suggests that the population have limited breeding success possibly caused by the elevated temperatures in the shallow lake (maximum depth 7 meters).

## Conclusion

Results from the present study show that temperature has a notable impact on the growth rates of two cold adapted Arctic charr populations. The populations investigated have been affected in different ways. Thus, our results show that the effect of warming on Arctic charr growth, depend on the magnitude of change and on local factors such as the morphometry of the system in question (river or lake), pathogens and food resources. Even though that this study is based on two localities with a limited sample size, the results are significant and indicate that the current increase in temperature is already having an impact on the Arctic populations.

## Methods

### Study sites

The study was carried out on Disko Island (69° 15'N, 53° 31'W) off the west coast of Greenland. Six study sites (four lakes and two rivers) were chosen known to have populations of Arctic charr. Data on wind, temperature, precipitation and albedo were obtained from records at the weather survey station at Qeqertarsuaq on Disko (1990 to 2001) and similar from the Danish Meteorological Institute's [18] climate station in Aasiaat on the west coast of Greenland approximately 65 km from Qeqertarsuaq (1981 to 1989).

### Sampling and treatment of material

Water temperatures were measured with depths at all study sites using a Hydrolab multisonde. Fish were caught in July and August 2004 with multi mesh gill net (Faarups Specialnet, Denmark) of a type proposed for monitoring use in Nordic countries (referred to as 'biological survey gill nets'; Nordic Norm) designed to catch representatively across a population. The nets were 40 meters long and composed of 12 mesh sizes: 29, 35, 5, 15.5, 24, 12.5, 8, 55, 10, 6.25, 19.5 and 43 mm. Sampling was carried out using randomized pelagic and littoral settings for each locality. The pelagic nets were placed parallel to the shore, while the littoral nets were set perpendicular to the shore. Specimens from the two rivers were obtained by angling (Nipisat Elv) or by hand nets and multi mesh gill nets (Røde Elv).

Populations suitable for climate studies were selected using principal component analysis (PCA) conducted in PCord-4 to examine the homogeneity following standard procedures detailed in [19] with respect to morphological quantitative characters (fork length, weight, age, mouth depth, mouth depth relative to fork length and condition factor [20]) and categorical characters (colour, sex, gonad maturity and stomach content).

Otoliths (sagittae) were sampled from all fish and subsequently mounted on a glass slide using resin at 140°C with the sulcus facing down and polished to enhance the annular ring structure. The age of the fish was estimated to the nearest year by counting of annual rings and by making transects to quantify the distance between annular rings using digital video microscopy with the software Image Pro Plus 5.0 (MediaCybernetics). Transects were placed as close to a downward angel of 90° to rostrum as possible in all examinations.

### Assessment of somatic growth

Measurements of otolith radius, fork length (F) and age at time of capture were used in constructing an Individual-Character matrix for each locality. We applied a recent model for growth back-calculation developed by Morita and Matsuishi [21] which takes into account that growth of fish (fork length) may be allometrically uncoupled from growth of their otolith. Multiple regression analyses were conducted using SYSTAT 8.0 (Systat Software Inc.) and back-calculated F-values were applied for calculation of growth rates using the simple relationship between body size at succeeding years:

*G*_*t *_= *F*_*t*+1 _- *F*_*t*_

where *G*_*t *_is the estimated growth rate at age *t*, and *F*_*t*+1 _and *F*_*t *_are fork lengths estimated by back-calculation at age *t *and *t+1*. To allow detection of signals from climate conditions on growth rates, the effect of ontogenetic development in every individual was subtracted from the *G*-values. We assumed a simple additive relationship between the effects of ontogenetic development and environmental conditions on the realized growth rate of the individual:

*G*_*t *_= *f*(*O*) + g(*E*)

where *O *is ontogenetic stage, *E *is the sum of environmental conditions and *f *and *g *are mathematical functions. Thus, to reveal the effects of climate, which are inherent in *E*, we subtracted the *O*-impact from the estimated *G*_*t *_and obtained what was termed annual growth rate residuals (*GR*_*t*_):

*GR*_*t *_= *G*_*t *_- *f*(*O*)

where the functional values of *O *were computed for each locality and for the total pooled data set by fitting a logarithmic function to the age-determined growth-tendency for the individual sample. This was done by logistic curve-fitting using the least-squares-method [22]. The resulting *GR*_*t *_values for each individual were assigned to the concurrent calendar year, thus obtaining the final time-individual matrix consisting of the growth rates for each individual. The mean growth tendency in the population was calculated annually and contrasted to variation in annual temperatures and precipitation.

### Modelling annual growth and climatic variation

Three annual levels of analysis were conducted. First, the constructed time series for annual fish growth and climatic variation, assessed by the parameters T_a_, T_s_, T_w _and P_a_, were depicted graphically for visual tendency interpretation. The Product Moment Correlation Coefficient r was calculated for opposed time series of fish growth and climate variables.

Secondly, multiple linear regressions were carried out (Systat Software Inc.) regressing *GR*_*t *_for each locality on T_a_, T_s_, T_w _or P_a_, respectively, incorporating one year delayed autoregressing (*GR*_*t*-1_):

*GR*_*t *_= α + *yGR*_*t*-1 _+ *x*_*t*_β

where *x*_*t *_is the mean climate parameter for temperature and precipitation and α, β and γ are regression constants.

Finally, to evaluate the impact of long-term climate change, we compared estimated growth curves between the two sampled time periods within each of the two landlocked populations. The growth curves formed a continuum from respectively 1982–1989 and 1995–2003. Data from 1990 and 2004 could not be used since the growth season had not finish at the time of sampling. The population means of the individual fork length estimates were tested by an ANOVA test [22] and plotted with corresponding standard errors of the estimate for each year. A statistical level of significance of 5% (p ≤ 0.05) was chosen for all analyses.

## Abbreviations

*F *= Fork length

F_em _= Fork length estimated means

*F*_*t *_= Calculated fork lengths at age t

*F*_*t*+1 _= Calculated fork lengths at age t + 1 year

*G *= Calculated growth rate

*GR*_*t *_= Growth rate residual

*GR*_*t*-1 _= Growth rate residual incorporating a factor of autoregressing

P_a _= mean annual precipitation

T_a _= mean annual temperature

T_s _= mean summer temperature

T_w _= mean winter temperature

## Authors' contributions

All authors contributed to the fieldwork. DMK, TRJ and RKL carried out analyses of otoliths, while DMK and TRJ carried out statistical analyses. KSC and MCF conceived of the study and DMK drafted the manuscript.

## Supplementary Material

Additional File 1 **Principal Component Analysis of individual fish morphology and stomach content, showing the two axes of greatest explanatory power**. Morphological quantitative characters included in analyses were fork length, weight, age, mouth depth, mouth depth relative to fork length and condition factor together with the following categorical characters colour, sex, gonad maturity and stomach content. Notice how Kangarssuk and Røde Elv individuals are closely scattered together indicating more homogeneous populations compared with the other sampled populations (Localities: 1 (k) = Kangarssuk; 2 (r) = Røde Elv; 3 (m/l) = Mellemsø/Langesø; 4 (p) = Porsilsø; 5 (n) = Nipisat. R^2 ^for vertical axis = 0.75 and horizontal axis = 0.23). Click here for file

## Figures and Tables

**Table 1:** Statistical values from multiple linear regression modelling between growth rate residuals, autoregressing and climate parameters from Kangarssuk and Røde Elv in 2004 and 1990.

**Kangarssuk 2004**	***P***	**Coefficient**	**S.E.**	**R**^2^
T_a_/GR_t-1_	0.53/0.99	9.2/0.01	12.98/0.46	0.15
T_s_/GR_t-1_	**0.04**/0.73	**15.15**/0.09	**4.35**/0.22	**0.80**
T_w_/GR_t-1_	**0.002**/0.15	-**25.88**/-0.07	**1.09**/0.03	**>0.99**
P_a_/GR_t-1_	0.24/0.77	1.58/0.13	1.09/0.39	0.41
T_s_/T_a_/GR_t-1_	**0.04/**0.18/0.66	**21.42**/-12.47/0.08	**4.35**/6.16/0.16	**0.94**
T_w/_T_a_/GR_t-1_	**0.04**/0.81/0.36	**-25.78**/0.38/-0.07	**1.5**/1.2/0.04	**>0.99**
P_a_/T_a_/GR_t-1_	0.27/0.43/0.69	0.084/10.58/0.181	0.06/10.71/0.4	0.61
T_s_/P_a_/GR_t-1_	**0.05**/0.14/0.36	**13.04**/0.05/0.17	**2.87**/0.02/0.14	**0.95**
T_w_/P_a_/GR_t-1_	**0.04**/0.46/0.29	**-24.72**/0.01/0.03	**0.94**/0.06/0.08	**>0.99**
T_s_/T_w_/GR_t-1_	0.96/0.07/0.42	0.142/-25.72/-0.07	2.09/2.83/0.05	>0.99
T_s_/T_a_/P_a_/GR_t-1_	0.09/0.24/0.21/0.31	17.76/-8.18/0.04/0.14	2.37/3.21/0.01/0.08	0.99

**Røde Elv 2004**	***P***	**Coefficient**	**S.E.**	**R**^2^

T_a_/GR_t-1_	**0.05**/11	**-13.34**/1.59	**-1.84**/1.22	**0.92**
T_s_/GR_t-1_	0.18/0.72	-5.75/-0.21	2.8/0.5	0.73
T_w_/GR_t-1_	0.99/0.59	0.1/-0.6	7.4/0.8	0.37
P_a_/GR_t-1_	0.30/0.66	0.03/-0.38	0.02/0.61	0.57
T_s_/T_a_/GR_t-1_	0.84/0.36/0.4	1.27/-15.65/1.9	4.95/9.97/1.39	0.92
P_a_/T_a_/GR_t-1_	0.59/0.23/0.31	0.01/-11.5/1.34	0.01/4.31/0.72	0.95
T_s_/P_a_/GR_t-1_	0.12/0.15/0.57	-4.99/0.02/-0.14	0.96/0.01/0.17	>0.99

**Kangarssuk 1990**	***P***	**Coefficient**	**S.E.**	**R**^2^

T_a_/GR_t-1_	0.7/0.48	-0.58/-0.36	1.39/0.46	0.2
T_s_/GR_t-1_	0.83/0.43	-0.89/0.4	3.97/0.45	0.17
T_w_/GR_t-1_	0.55/0.42	-0.33/-0.39	0.51/0.43	0.24
P_a_/GR_t-1_	0.12/0.18	-0.1/-0.54	0.05/0.33	0.57
T_s_/T_a_/GR_t-1_	0.92/0.77/0.58	0.77/-0.78/0.34	6.8/2.42/0.55	0.2
T_w/_T_a_/GR_t-1_	0.54/0.63/0.41	-1.22/2.51/-0.55	1.77/4.73/0.56	0.31
P_a_/T_a_/GR_t-1_	0.07/0.21/0.09	-0.17/2.07/-0.8	0.06/1.31/-0.8	0.77
T_s_/P_a_/GR_t-1_	0.53/0.15/0.22	2.48/-0.12/-0.56	3.52/0.06/0.36	0.63
T_w_/P_a_/GR_t-1_	0.07/0.17/0.9	-0.21/1.02/-0.73	0.07/0.56/0.29	0.8
T_s_/T_w_/GR_t-1_	0.91/0.63/0.5	0.68/-0.38/-0.38	5.25/0.7/0.5	0.24
T_s_/T_a_/P_a_/GR_t-1_	0.88/0.39/0.16/0.19	-0.78/2.3/-0.18/-0.82	4.53/2.11/0.08/0.42	0.77
T_w_/T_a_/P_a_/GR_t-1_	0.64/0.9/0.16/0.19	0.83/0.46/-0.21/-0.75	1.5/3.27/0.09/0.39	0.8
T_s_/T_w_/T_a_/GR_t-1_	0.65/0.51/0.56/0.43	-5.91/-2.47/7.24/-0.9	11.1/3.1/10.4/0.89	0.39
T_s_/T_w_/P_a_/GR_t-1_	0.75/0.31/0.14/0.17	1.19/0.94/-0.21/-0.72	3.25/0.7/0.09/0.34	0.81
T_s_/T_w_/T_a_/P_a_/GR_t-1_	0.74/0.66/0.79/0.36/0.58	4.51/2.19/-3.56/-0.25/-0.54	10.6/3.74/10.4/0.15/0.70	0.83

**Røde Elv 1990**	***P***	**Coefficient**	**S.E.**	**R**^2^

T_a_/GR_t-1_	0.3/0.62	0.55/-0.25	0.46/0.46	0.26
T_s_/GR_t-1_	0.47/0.99	1.12/0.002	1.41/0.49	0.14
T_w_/GR_t-1_	0.56/0.66	0.14/-0.27	0.22/0.56	0.1
P_a_/GR_t-1_	0.6/0.91	-0.02/0.07	0.03/0.56	0.08
T_s_/T_a_/GR_t-1_	0.82/0.51/0.64	-0.7/0.76/-0.37	2.86/1.01/0.71	0.28
T_w/_T_a_/GR_t-1_	0.25/0.17/0.68	-0.78/2.29/0.24	0.54/1.28/0.53	0.57
P_a_/T_a_/GR_t-1_	**0.05**/**0.03**/0.78	**-0.06**/**1.19**/0.08	**0.02**/**0.32**/0.27	**0.83**
T_s_/P_a_/GR_t-1_	0.1/0.11/0.22	3.25/-0.06/0.72	1.37/0.03/0.47	0.68
T_w_/P_a_/GR_t-1_	0.09/0.09/0.72	0.55/-0.07/-0.15	0.22/0.03/0.38	0.7
T_s_/T_w_/GR_t-1_	0.72/0.97/0.98	1.05/0.01/-0.02	2.64/0.40/0.88	0.14
T_s_/T_a_/P_a_/GR_t-1_	0.54/0.23/0.09/0.54	1.2/0.9/-0.06/0.32	1.61/0.53/0.02/0.43	0.87
T_w_/T_a_/P_a_/GR_t-1_	0.84/0.31/0.21/0.77	-0.12/1.42/-0.05/0.14	0.54/1.06/0.03/0.40	0.84
T_s_/T_w_/T_a_/GR_t-1_	0.72/0.34/0.72/0.92	-1.07/-0.81/2.68/0.08	2.63/0.64/1.78/16.0	0.60
T_s_/T_w_/P_a_/GR_t-1_	0.27/0.26/0.09/0.54	2.05/0.36/-0.08/0.34	1.36/0.23/0.02/0.45	0.86
T_s_/T_w_/T_a_/P_a_/GR_t-1_	0.7/0.92/0.79/0.38/0.7	1.37/0.1/0.68/-0.07/0.31	2.68/0.8/1.97/0.05/0.61	0.87

Statistical significant results are shown in bold (T_a _= Mean annual temperature; T_s _= Mean summer temperature; T_w _= Mean winter temperature; P_a _= Mean annual precipitation; *GR*_*t*-1_: autoregression).
