# Supplementary figures and images for: Inter-annual growth of Arctic charr (Salvelinus alpinus, L.) in relation to climate variation
Source: BMC Ecol. 2006 Aug 27;6:10. doi: 10.1186/1472-6785-6-10 (PMC1560112; doi:10.1186/1472-6785-6-10)

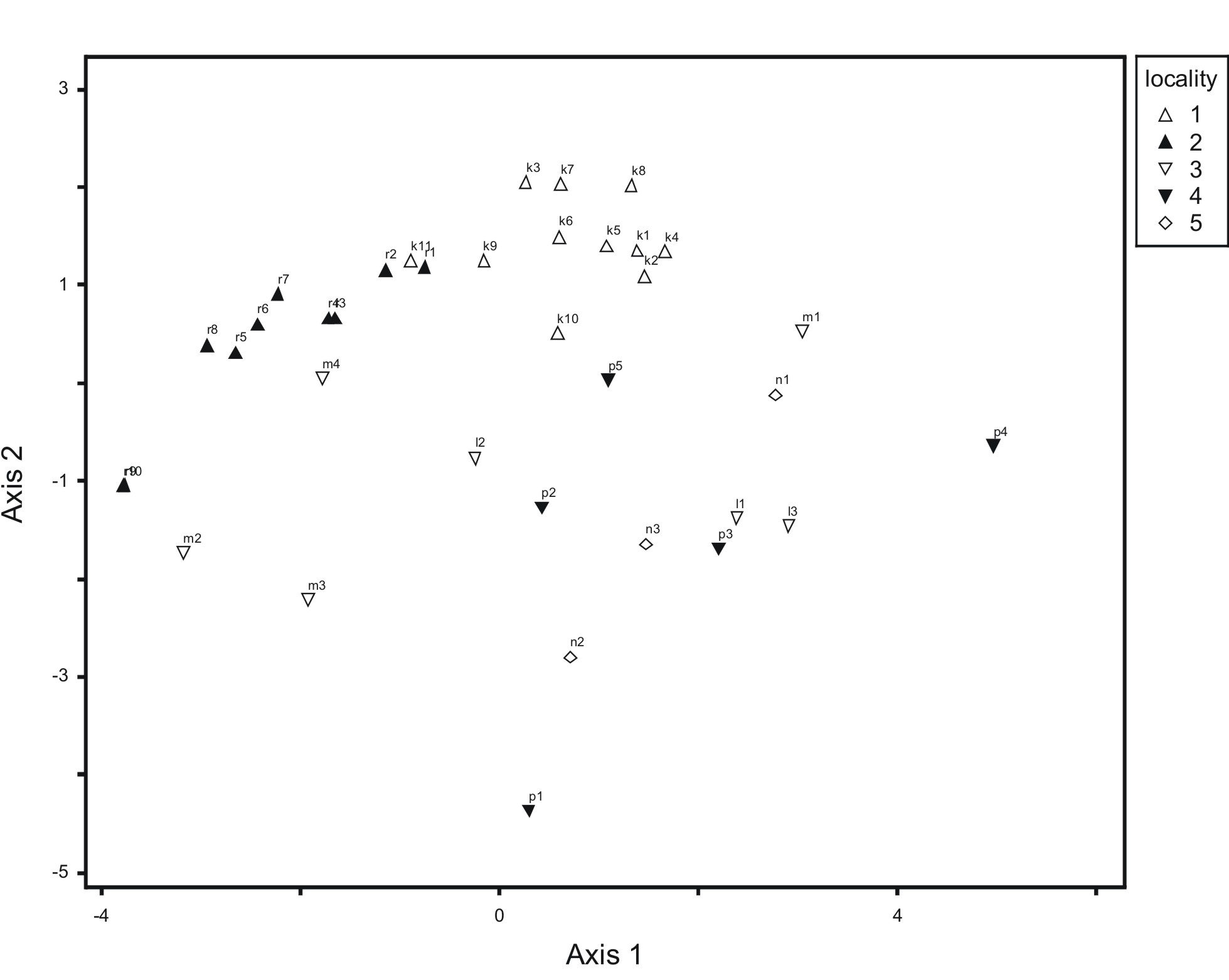

Supplement: Additional File 1 — Principal Component Analysis of individual fish morphology and stomach content, showing the two axes of greatest explanatory power. Morphological quantitative characters included in analyses were fork length, weight, age, mouth depth, mouth depth relative to fork length and condition factor together with the following categorical characters colour, sex, gonad maturity and stomach content. Notice how Kangarssuk and Røde Elv individuals are closely scattered together indicating more homogeneous populations compared with the other sampled populations (Localities: 1 (k) = Kangarssuk; 2 (r) = Røde Elv; 3 (m/l) = Mellemsø/Langesø; 4 (p) = Porsilsø; 5 (n) = Nipisat. R2 for vertical axis = 0.75 and horizontal axis = 0.23). [file 1472-6785-6-10-S1.doc]
